# A transducer positioning method for transcranial focused ultrasound treatment of brain tumors

**DOI:** 10.3389/fnins.2023.1277906

**Published:** 2023-10-11

**Authors:** Penghao Gao, Yue Sun, Gongsen Zhang, Chunsheng Li, Linlin Wang

**Affiliations:** ^1^Artificial Intelligence Laboratory, Shandong Cancer Hospital and Institute, Shandong First Medical University and Shandong Academy of Medical Sciences, Jinan, Shandong, China; ^2^Department of Radiation Oncology, Shandong Cancer Hospital and Institute, Shandong First Medical University and Shandong Academy of Medical Sciences, Jinan, Shandong, China; ^3^Department of Biomedical Engineering, Shenyang University of Technology, Shenyang, Liaoning, China

**Keywords:** transcranial focused ultrasound, transducer positioning, acoustic field, FWHM, sonication-induced, thermal effects

## Abstract

**Purpose:**

As a non-invasive method for brain diseases, transcranial focused ultrasound (tFUS) offers higher spatial precision and regulation depth. Due to the altered path and intensity of sonication penetrating the skull, the focus and intensity in the skull are difficult to determine, making the use of ultrasound therapy for cancer treatment experimental and not widely available. The deficiency can be effectively addressed by numerical simulation methods, which enable the optimization of sonication modulation parameters and the determination of precise transducer positioning.

**Methods:**

A 3D skull model was established using binarized brain CT images. The selection of the transducer matrix was performed using the radius positioning (RP) method after identifying the intracranial target region. Simulations were performed, encompassing acoustic pressure (AP), acoustic field, and temperature field, in order to provide compelling evidence of the safety of tFUS in sonication-induced thermal effects.

**Results:**

It was found that the angle of sonication path to the coronal plane obtained at all precision and frequency models did not exceed 10° and 15° to the transverse plane. The results of thermal effects illustrated that the peak temperatures of tFUS were 43.73°C, which did not reach the point of tissue degeneration. Once positioned, tFUS effectively delivers a Full Width at Half Maximum (FWHM) stimulation that targets tumors with diameters of up to 3.72 mm in a one-off. The original precision model showed an attenuation of 24.47 ± 6.13 mm in length and 2.40 ± 1.42 mm in width for the FWHM of sonication after penetrating the skull.

**Conclusion:**

The vector angles of the sonication path in each direction were determined based on the transducer positioning results. It has been suggested that when time is limited for precise transducer positioning, fixing the transducer on the horizontal surface of the target region can also yield positive results for stimulation. This framework used a new transducer localization method to offer a reliable basis for further research and offered new methods for the use of tFUS in brain tumor-related research.

## 1. Introduction

Brain tumors have been represented as the second most frequent etiology in patients with focal seizures, mostly in the temporal lobe (Slegers and Blumcke, 2020). In the early stage of temporal lobe tumors, there are no obvious clinical symptoms, but as the disease progresses, the tumor increases in size and is often accompanied by temporal lobe seizures. Due to the complexity of the functional areas of the temporal lobe, surgery was the choice in the past, but the considerable risks were carried by surgical resection and a high risk of long-term side effects owing to the specificity of the tumor itself and effects including surgical complications, radioactive neurotoxic effects and chemotherapy-induced debility (Vargo, 2011). Related studies by Monje have revealed that tumor cells have the ability to manipulate and respond to signals produced by neurons, which aid their growth. Additionally, these cells have been observed to grow faster in the vicinity of highly active neurons, suggesting a link between tumor cells and neurons (Svider et al., 2017; Venkatesh and Monje, 2017). Despite the considerable advancements in therapeutic approaches to other malignancies, the treatment of brain tumors has not improved significantly in recent decades (Venkatesh et al., 2015).

Transcranial focused ultrasound (tFUS) is a non-invasive neuromodulation tool that focuses and delivers ultrasound energy to localized areas of the brain to modulate neuronal activity (Min et al., 2011; Lescrauwaet et al., 2022; Armstrong et al., 2023). The subsequent biological models (Konofagou et al., 2012; Zou, 2020; Yan et al., 2021; Cain et al., 2022) have found a significant reduction in the number and duration of seizures after low-intensity tFUS stimulation, providing important experimental evidence for clinical translation. Preliminary evidence that tFUS has a role in modulating the therapeutic and tumor suppressor activity of abnormal neural networks. Compared to other neuromodulation techniques of tFUS, transcranial magnetic stimulation (TMS) and transcranial direct-current stimulation (tDCS) have garnered approval for the treatment of select neurological disorders, based on their demonstrated effectiveness (Klomjai et al., 2015; Iglesias, 2020; Cappon et al., 2022; Marder et al., 2022). Compared to magnetic fields, acoustic fields are controllable and acoustic waves can be focused on a point in the deep brain like light (Bystritsky et al., 2011; Kubanek, 2018). Therefore, tFUS has a broader research prospect with its higher spatial precision and regulation depth.

In previous studies of single-element transducers, the transducer had to be manually repositioned to achieve a nearly perpendicular angle of orientation toward the target area along the skull surface, a process which often resulted in unintentional errors and variations that compromised the intended focus of the acoustic beam (Yoon et al., 2018). The numerical modeling of tFUS treatments remains complex, mainly owing to the non-linearity arising along ultrasound propagation, the anatomical complexity, the heterogeneity of the medium, the inter-individual variability of the properties of the medium, *in vivo* tissue movements and the correlation between temperature and biological damage (Grisey et al., 2016). Heger et al. have simulated sonication acoustic fields by modifying transducer components to evaluate the potential application of targeted FUS systems in animals sensitive to cerebral hemorrhage (Grudzenski et al., 2022). Park’s numerical approach based on subject-specific sonication beamline simulations to find the optimal configuration of ultrasound probes for sonication stimulation has demonstrated that the specific tFUS framework he developed can be effectively used for human neuromodulation studies (Park et al., 2022). Shen’s study on the effect of low-frequency sonication (21 kHz, 26 mW/cm^2^, 40% duty cycle, 3 min) combined with microbubbles on nude mice with subcutaneous prostate adenoma showed a loss of blood flow signal at the tumor and a decrease in tumor volume (Shen et al., 2014).

Sonication of 200 kHz to 1.5 MHz are mostly used in clinical practice, among which low and medium frequency sonication of 100 kHz to 1 MHz can be effectively applied in tissue destruction and neuromodulation, with the intensity of 30 to 500 mW/cm^2^ can penetrate the skull to stimulate the functional brain region and regulate neuronal functions through mechanical effects (Krishna et al., 2018; Maresca et al., 2018). Low-intensity tFUS destroys tumor blood vessels by increasing tissue permeability to promote apoptosis and inhibit tumor growth and proliferation for the purpose of anti-tumor. However, the acoustic parameters of the skull are strongly inhomogeneous, which have been demonstrated that sonication penetration through thick and dense skull and focusing on a predetermined point is very tricky. Owing to the presence of the skull in sonication stimulation, the offset between the focal point and the preset intracranial target region caused by the acoustic properties between different media (i.e., speed of sound, density, and attenuation coefficient). Accurate prediction of the sonication path of stimulation has also become an international conundrum (Servick, 2020).

The absorption of acoustic energy by biological tissues leads to an increase in temperature, resulting in increased excitability of neurons. Sonication of a certain intensity causes transient high temperature in the target region of the brain through other effects, leading to protein denaturation and tissue coagulation necrosis, permanently damaging the lesion and modulating the neural network. Denaturation of tissue temperature occurs at 42°C (Dickson and Calderwood, 1980). At 43 to 60°C, tissue damaged at an exponential rate, reaching 47°C leads to cancellous bone necrosis (Augustin et al., 2012), and reaching 50°C causes necrosis of cardiac muscle cells, producing irreversible tissue damage (Darrow, 2019).

In this study, we performed numerical simulation of sonication stimulation penetration through the skull with different parameters based on the effective cavitation effect stimulation parameters obtained from the research and located the optimal position of the transducer. The new RP (Radius Positioning) method performed the selection of the effective range of stimulation based on the parameters of the transducer, which was not available in other studies. Acoustic field simulation and calculation of sonication stimulation paths were performed based on transducer positioning results, with the effective error of FWHM (Full Width at Half Maximum) obtained, which provided a numerical theoretical reference for achieving accurate and effective neuromodulation (Schwenke et al., 2017). We also verified the effectiveness of transducer positioning by simulating each directional angle of the control group. The transducer after positioned was non-serendipitous compared to the given position in other studies, while the temperature field simulation with added thermal effects was able to verify the safety feasibility of this study, showing that only small temperature changes are produced by sonication at low intensities, providing a new simple and effective numerical method for the application of the tFUS in clinical neuromodulation.

## 2. Materials and methods

Firstly, a 3D skull model was reconstructed from the binarized CT images in this study. Secondly, the transducer matrix and transducer positioning of matrix were performed by acoustic pressure (AP) simulations based on the settings of target region and the parameters of the transducer. Successively, the distribution of the sonication beam in the skull was simulated and the distance of the stimulation and directional angles were calculated based on the transducer positioning results. The correctness of transducer positioning was proven according to the simulation results by adding sonication stimulus with different directional angles at the target region. Finally, we demonstrated the safety of whole process by predicting the acoustic temperature field of the thermal effects.

### 2.1. Data acquisition

The data of this research were the CT images of the brain collected in Shandong Cancer Hospital and Institute with the size of 512×512×245 , including the CT data and contour information of the patient, which was used to obtain the 3D skull anatomical structure. The study was conducted in accordance with the Declaration of Helsinki, and the protocol was approved by the Ethics Committee of Shandong First Medical University Affiliated Cancer Hospital (Approval ID: SDTHEC2023002013). Written informed consent for participation was not required for this study in accordance with the national legislation and institutional requirements.

### 2.2. Skull model

**Figure 1:**
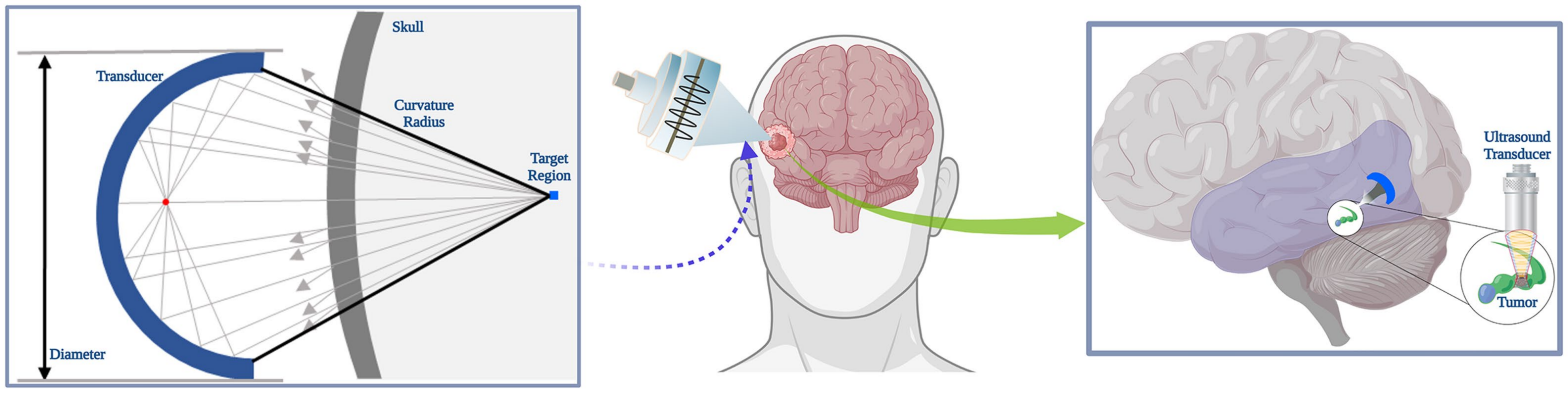
Schematic of sonication simulation. A simple schematic diagram of tFUS stimulation effects in brain tumor.

The 3D CT images were all resampled to a spatial size of 1mm×1mm×1mm assuring that the same physical size information contained within the voxel. The resampled CT data (Hounsfield Unit) were extracted using 3D Slicer at a threshold intensity of H=100HU to filter other tissues, which was reconstructed into a 3D skull model after binarization by MATLAB (R2019b, MathWorks Inc., Natick, MA, USA). The skull model was established for the subsequent application of temporal lobe hippocampal acoustic field simulation, therefore the model removed the parietal bone for better observation of the 3D sonication beam distribution. A simple simulation schematic under the transverse plane was depicted in Figure 1, where the target region was located.

### 2.3. Sonication setup

#### 2.3.1. Ultrasound

**Figure 2:**
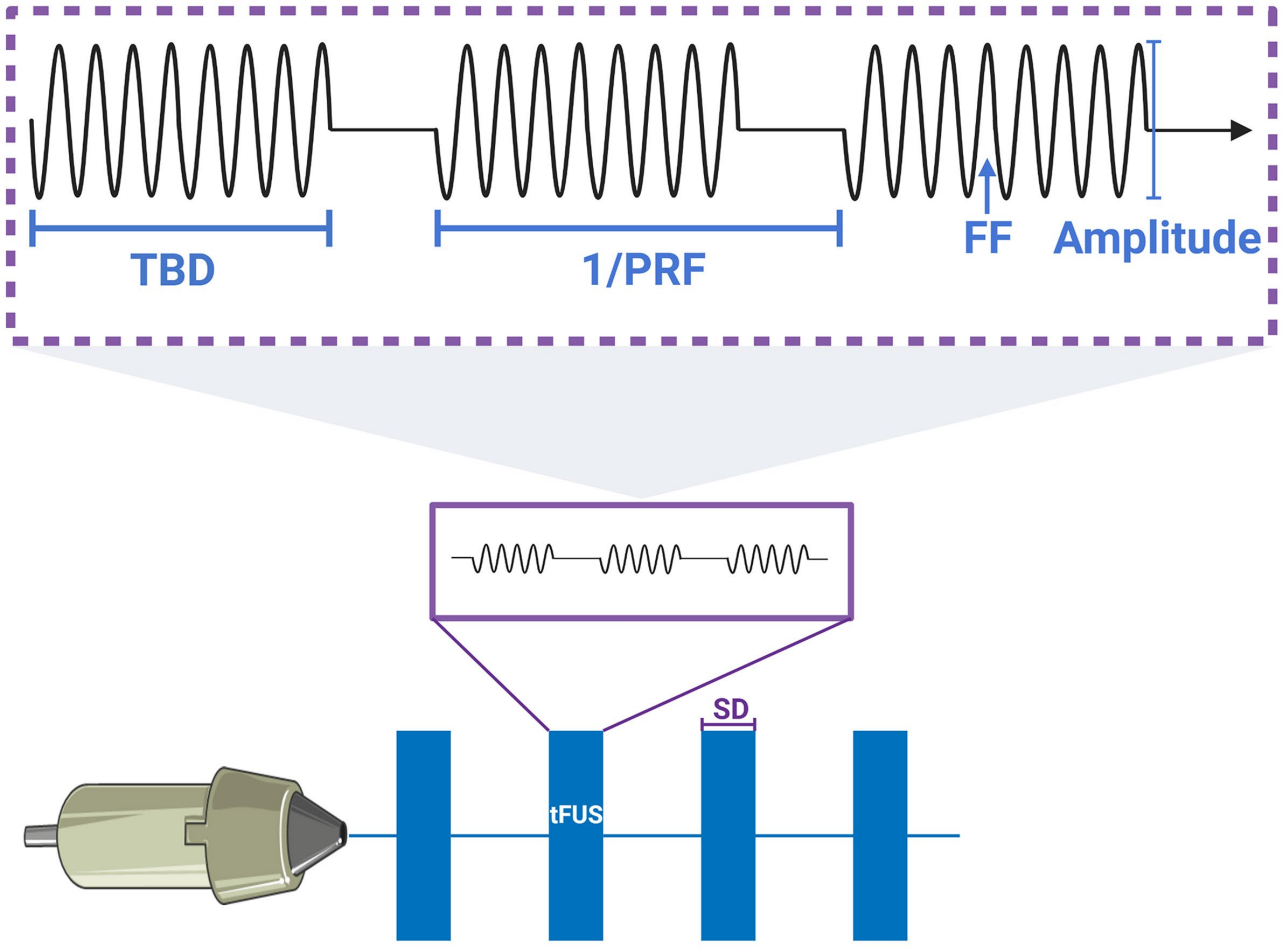
Typical schematic and parameters of pulsed sonication sequences for neuromodulation. The explanation of parameters that SD was sonication duration; PRF was pulse repetition frequency, the 1/PRF was the one cycle; TBD was tone burst duration; FF was fundamental frequency.

In this study, the sonication parameters were selected according to the function of the Olympus Scientific Solutions (Olympus America Inc., PA, US) V301-SU ultrasound probe. The optimal parameters for the simulation of low-frequency sonication were conducted at a frequency of 500 kHz and with a pressure level of 1 MPa, while it has been shown that soft tissue does not play a significant role at this frequency and has relatively deep penetration and fine spatial resolution (Mueller et al., 2017). At the time of this study, no prior literature existed on the applications of radius filtering in the positioning of neuromodulations in focused ultrasound. Considering that most focused ultrasound systems were used for cellular ablation, the optimization of parameters was warrantable. We have carefully selected the parameters in this study, ensuring that they remained well below the U.S. Food and Drug Administration (FDA) guidelines for diagnostic sonication (mechanical index $\left(MI\right)\leq 1.9$ , spatial-peak pulse-average intensity $\left(I_{\mathrm{SPPA}}\right)\leq 190W/cm^{2}$ ) after transcranial transmission. The classical frequency of focused ultrasound, 250 kHz, 690 kHz, and 1.1 MHz were also chosen in sonication simulation by considering previous studies regarding FUS-induced BBB disruption (Kinoshita et al., 2006; Cho et al., 2016; Ilovitsh et al., 2018; Huh et al., 2020; Park et al., 2021). The F-number of the transducer was calculated as the ratio of the radius of curvature to the diameter of the transducer. Specifically, a focused radius of curvature of 30 mm and a diameter of 25 mm (which was the nominal wafer size) were selected. Additionally, the diameter of the transducer (which was 31 mm) was considered a control parameter in this study. The numerical parameters were chosen a fundamental frequency of 500 kHz, an excitation AP of 1 MPa, a stimulation duration of 500 ms, a single tone burst duration (TBD) of 200 μs, a PRF of 300 Hz (150 cycles) and a duty cycle (DC) of 6% for the simulation of the AP and sonication-induced thermal effects. We posited that low-intensity sonication, when employed with lower duty cycles, does not induce substantial thermal effects, thus effectively suppressing the excitability of tumor cells. A typical schematic and parameters of sonication pulse sequences used for neuromodulation were shown in Figure 2. The sonication sequences in this schematic were generated by programming the MATLAB toolbox.

#### 2.3.2. Acoustic property

The soft tissues mainly supported the propagation of compressional waves and hardly support shear waves, while the skull was capable of transmitting both waves, but the shear waves were very weak when low-frequency sonication penetrating the skull, therefore the effect of shear waves was not considered in this study. The results of relevant acoustic characterization studies demonstrate that the nonlinear parameters BonA were between 5 and 11 in most soft tissues, while solids have a negligible ability to generate nonlinearities (Slezak et al., 2022; Armstrong et al., 2023). Given the highly similar properties of tissue fluid and water, the skull model was considered to be immersed in water to couple it to the ultrasound source. Furthermore, the brain tissues were approximated as being water to lessen the model complexity and computational time. The nonlinear parameter BonA was set to a value of 5.2 (Bjørnø, 2010). Meanwhile, owing to the homogenization of the model grid, the effect of pulsation of cerebrospinal fluid flow was avoided. The Courant Friedrichs Lewy (CFL) in this study was a constant 0.3 and the power law absorption exponent was set to a constant 1.51 (Duck, 1990).

Owing to differences in the acoustic properties of the skull, the feasibility of numerical calculation of tFUS attenuation varies among individuals (Jing and Lindsey, 2021). The acoustic properties of the skull model were calculated from the porosity (𝜓) of the CT images at a given ultrasound frequency by eq. (1). Based on the results of high-resolution CT images simulation studies showed that the attenuation of tFUS was highly dependent on the porosity of the individual skull (Mueller et al., 2017). The porosity was used to calculate the absorption of sonication, which lead to an increase with high porosity in simulated absorption values. The compressive sound velocity 𝑐, density 𝜌, and attenuation coefficient 𝛼 of the skull were calculated by eqs. (2)–(4) as shown in Table 1, where the maximum and minimum skull compression attenuation were referred to in the literature (Mueller et al., 2017).


(1) $$\psi =1-\frac{H}{1000}$$

(2) $$c_{\mathrm{skull},c}=c_{\mathrm{water}}\psi +c_{\mathrm{bone},c}\left(1-\psi \right)$$

(3) $$\rho_{\mathrm{skull}}=\rho_{\mathrm{water}}\psi +\rho_{\mathrm{bone}}\left(1-\psi \right)$$

(4) $$\alpha_{\mathrm{skull},c}=\alpha_{\min,\mathrm{skull},c}+\left(\alpha_{\max,\mathrm{skull},c}-\alpha_{\min,\mathrm{skull},c}\right)\psi^{0.5}$$

**Table 1:** Acoustic parameters.

Speed (m/s)	Density (kg/m^3^)	Attenuation coefficient (Np/MHz·m)
$c_{\mathrm{water}}$ =1,482	$\rho_{\mathrm{water}}$ =1,000	$\alpha_{\mathrm{water}}$ =3.48 × 10^−4^
$c_{\mathrm{bone}}$ =3,100	$\rho_{\mathrm{bone}}$ =2,200	$\alpha_{\min,\mathrm{skull}}$ =21.5, $\alpha_{\max,\mathrm{skull}}$ =208.9 (Connor, 2005)

### 2.4. Acoustic pressure simulation

The acoustic simulation was performed based on the k-space pseudospectral method of MATLAB k-Wave toolbox. The linear wave equation resolved by numerical simulation had been proven to be a reliable method for calculating sonication beams in complex structures (Legon et al., 2018). The k-wave toolbox, an open-source software package highly regarded in the field of acoustic wave simulations, has been developed to effectively model and analyze the intricate behavior of acoustic waves within complex media. Its versatile functionality encompasses a broad range of applications, including ultrasound imaging, ultrasound therapy, and bioacoustics (Treeby and Cox, 2010). The domain of the acoustic simulation performed in this study was 256×256×256 in the 3D model represented the skull model with 256mm×256mm×256mm . In view of the high computational time cost of this precision model, this study introduced a twofold-precision model with a size of 128×128×128 to expedite the computation process. Successively, the 31 mm transducer was selected in the domain of 210×210×210 . For simplicity, the target regions in this study were assumed to be a point instead of a 3D target region volume. The volume stimulated in the target region was observed through the 3D acoustic field.

### 2.5. Radius positioning method

The approach taken by some researchers in defining a target region solely within the region under the skull vault and assessing the acoustic field effect at several extracranial points perpendicular to the skull vault is inherently restrictive. Such an approach fails to adequately identify an optimal location for the practical implementation of target region definition in tFUS investigations. To ascertain the effective range of transducer centroid arrays, we developed a RP algorithm to refine the options and enhance computational efficiency. This algorithm narrowed down the transducer matrix to a specific range by excluding areas inside the skull and outside the circle formed by the radius of curvature, as determined by the established transducer curvature. Additionally, to prevent contact with the skull, another component of the RP algorithm identified points that were too close to the skull layer, thus ensuring safe transducer placement. The underlying concept of the RP algorithm is outlined as follows.

In the skull model with inhomogeneity, the center of the tumor in the hippocampal region of the medial temporal lobe was selected as the target region. With the target region selected as the center, a ball was outlined and the point matrix in the intersection of the ball and the skull layer was screened. The center of the model was established by identifying the midpoint of the skull profile at the extremes on the coordinate axis. The average distance from the model center to the intersecting skull layer was meticulously calculated. The points, outside the model but within the ball having a distance greater than the calculated average from the center point, were selectively assembled into the transducer matrix. Simultaneously, the algorithm effectively removed the center points that may correspond to the contact area between the transducer and the skull layer, accounting for the size of the transducer. The calculated transducer matrix will be utilized in the subsequent stage of transducer positioning.

### 2.6. Transducer positioning

**Figure 3:**
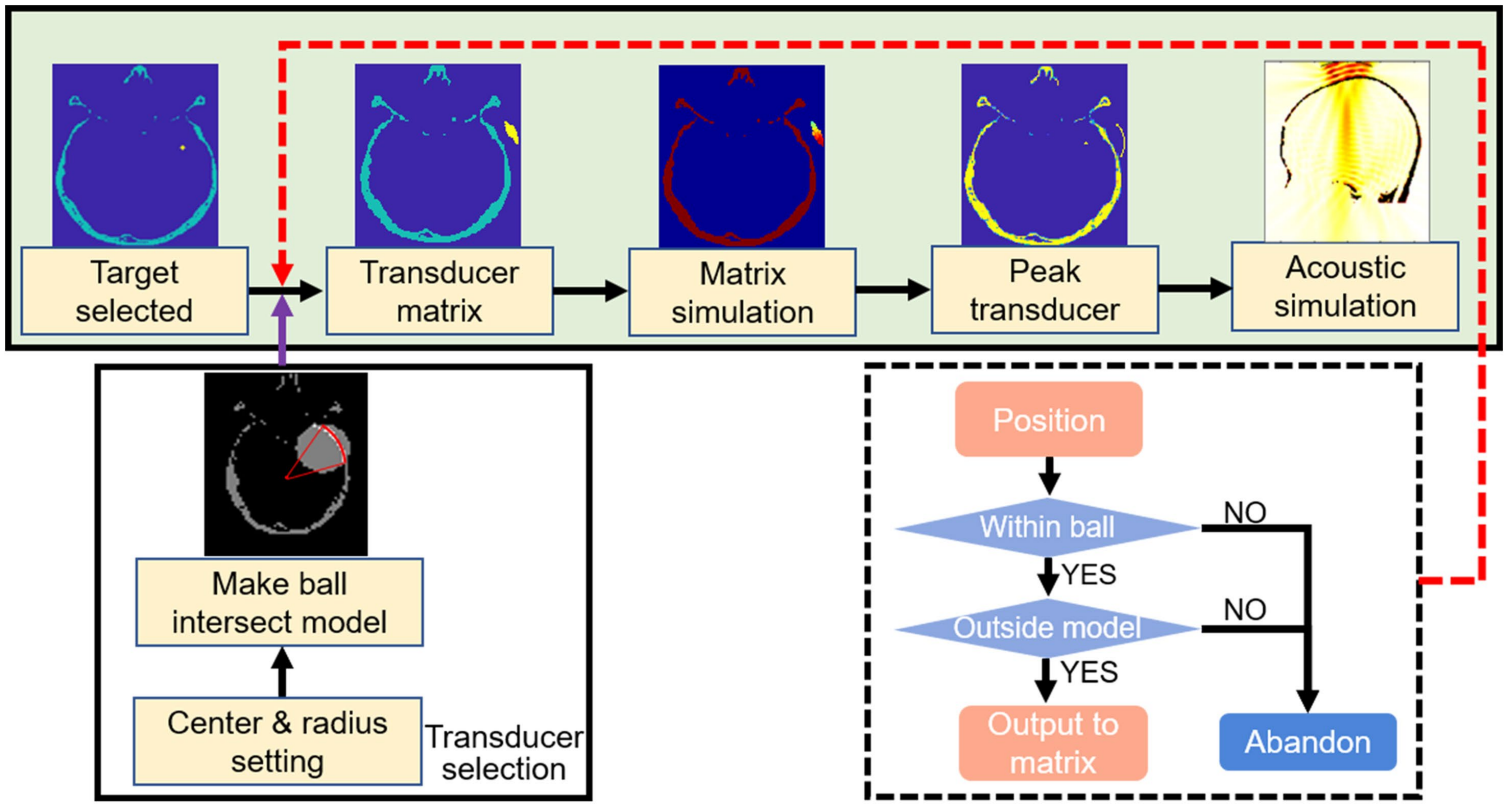
Flow chart of transducer positioning. Selected the center of tumor as the target region. A ball was outlined according to the parameters of ultrasound probe. The transducer matrix was selected after the outlined ball intersected with the skull layer. The transducer was positioned by simulated the transducer matrix and selected the point with peak pressure. The acoustic field of sonication penetrated the skull was simulated.

The transducer matrix was subjected to acoustic pressure simulation to screen the center of the transducer, as the transducer positioning flow chart shown in Figure 3. With the uniform attenuation medium in an inhomogeneous skull, the precise location of the transducer point was determined as the region with the lowest level of attenuation after penetrating the skull. Based on the conditions of transducer positioning, the fraction of the control group cases greater than the directional angle of 45 degrees neglected the effect of weak shear waves. Acoustic field simulation of the target region was performed after transducer positioning, with the path from the center to the target region screened as the best stimulation path, the vector angles in each direction were calculated as well as the stimulation distance. Related studies have confirmed that sonication-induced neuromodulatory effects appear within the FWHM (Lee et al., 2015, 2016; Legon et al., 2018). The contours of FWHM at different frequencies were depicted after the normalization of the acoustic field.

Sonication stimulations from various directional angles (i.e., 0°, 30°, 45°, 60°) were administered to the target region as a simulated control group, with the transducer positioned extracranially along the directional path and the curvature radius as stimulation distance. Directional angles in the transverse plane and coronal plane were explored in the control group.

### 2.7. Thermal simulation

The open-source k-Wave toolbox was also used to solve the Pennens’ bioheat equation (Treeby and Cox, 2010; Legon et al., 2018), as the peak temperature of thermal effects generated in the tissue during sonication stimulation was simulated by k-Wave (Xu et al., 2020). The spatial conditions (i.e., simulation domain and grid size) of the thermal simulation were the same as acoustic simulation. Uniform segmentation of inhomogeneous brain tissue through a spatial grid of the simulation domain. The thermal effect simulation not only added the medium properties related to thermal diffusion, as the value of the skull [specific heat =1300J/kg/K , thermal conductivity $=1.16\times 10^{-2}\;W/\mathrm{cm}/{}^{\circ}C$ (Nelson and Nunneley, 1998)] and water [specific heat =4178J/kg/K , thermal conductivity =0.54W/cm/°C (Duck, 1990)] were assigned, but also explored the temperature change under sonication stimulus through the parameters of ultrasound source (i.e., opening time, closing time and time step),while the temperature field of thermal effects was also monitored in real-time. The stimulation parameters were a duration of 500 ms and a duty cycle of 6% for the sonication interval. The initial tissue temperature in the simulated cranium was set at 38.5°C, which was the experimentally derived average value of the relevant studies (Rzechorzek et al., 2022).

## 3. Results

### 3.1. Acoustic pressure On target region

**Figure 4:**
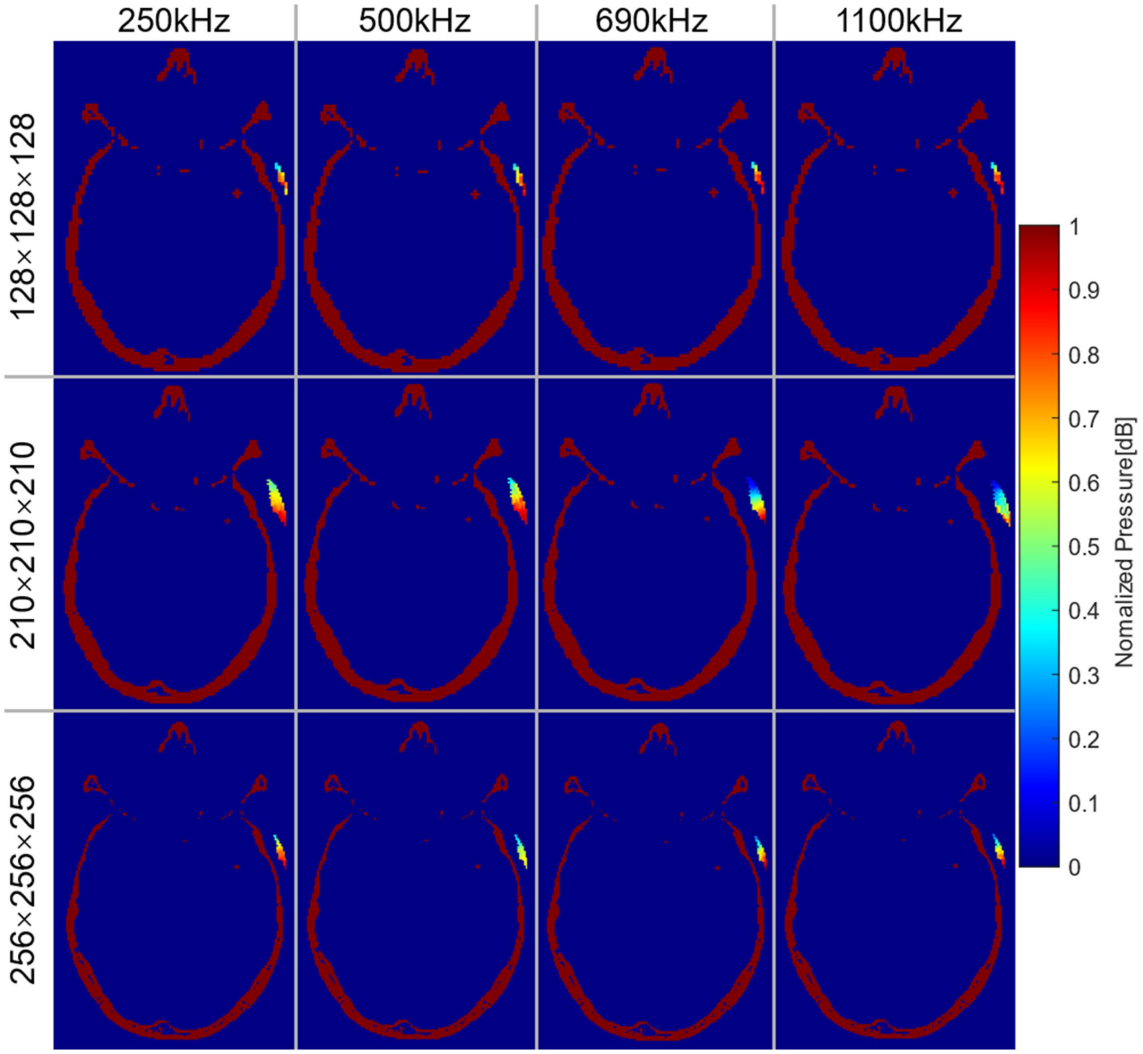
Normalized AP distribution of transducer matrix at the transverse plane of the target region. Each row represented the results under different precision models. Each column represented the results at different frequencies.

**Figure 5:**
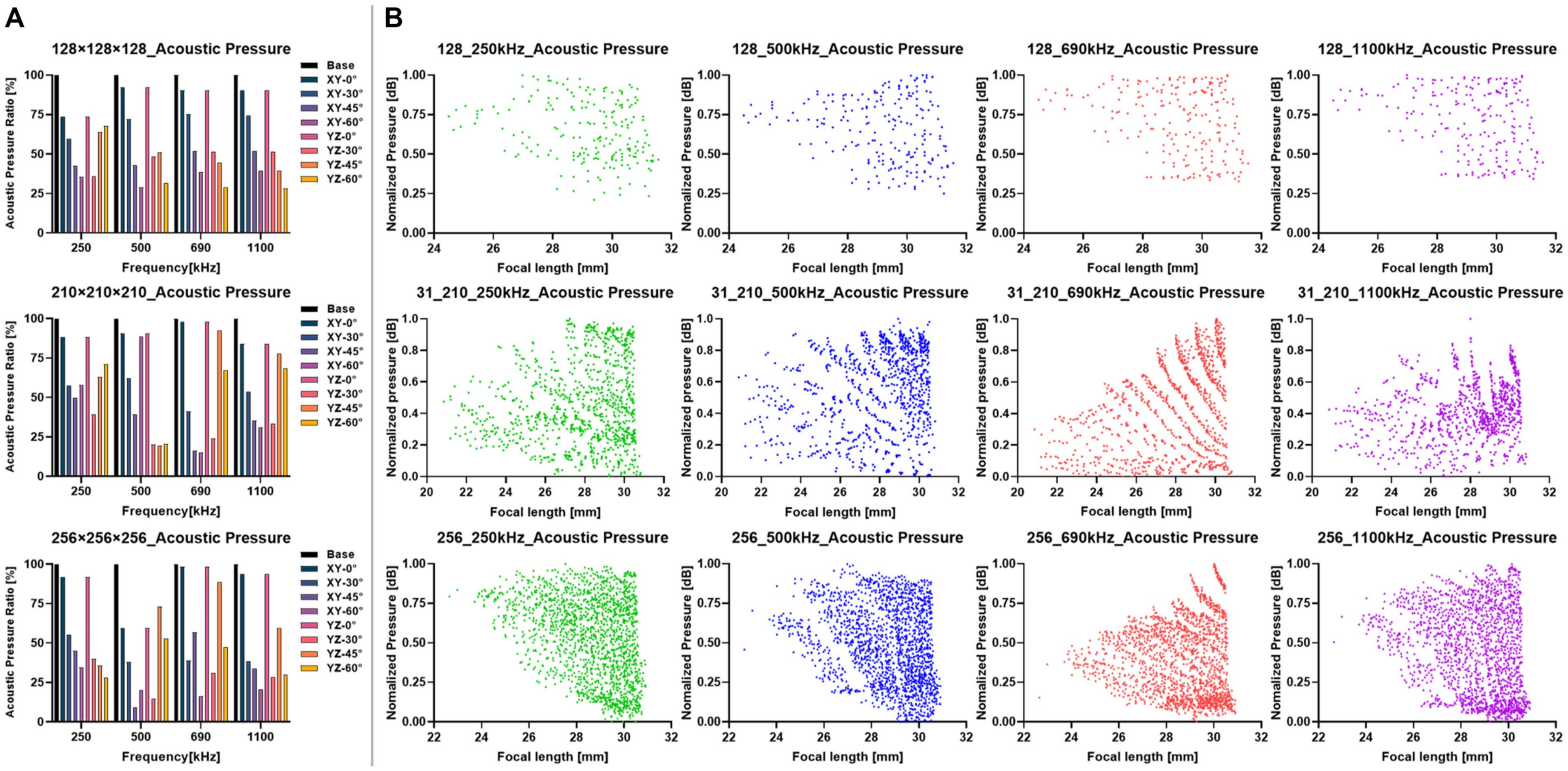
Results of the sonication stimulations at different transducer positions. **(A)** Comparison of acoustic pressure in the target region at each directional angle with different precision models (i.e., 128×128×128 , 210×210×210 , 256×256×256 ). The directional angle of XY was at the transverse plane, the YZ was at the coronal plane. The Base was the AP of the positioned transducer. **(B)** Distribution of AP at the distance from the transducer to the target region. Each row represented the results under different precision models. Each column represented the results at different frequencies.

Different frequencies of sonication were performed for positioning under different precision models. Figure 4 illustrated the normalized AP distribution results of the transducer matrix in the transverse plane of the target region. The highest AP values were predominantly concentrated at the lower end of the matrix. Figure 5B illustrated the distribution of simulated AP at various frequencies, along with the corresponding distance from the transducer to the target region, in different precision models. The transducer matrix, chosen using the RP algorithm, exhibited comparable AP distributions following sonication stimulation at various frequencies, with a notable concentration of points approximately 30 mm away from the target region. It can be proved that the AP stimulated on the target region was less influenced by the distance between the transducer and the target region. This was supported by Figure 4, which demonstrated a relationship between the direction angle applied to the target area and the resulting AP. As the precision of the model improved, the effectiveness of the RP algorithm was more obviously explored. The accuracy of the algorithm exceeded 99.50% for models with a precision of 1 mm/grid. It was noteworthy that the AP distribution at 690 kHz differed from the other three frequencies examined, as the peak AP point from the transducer remained approximately constant at a distance of approximately 30 mm from the target region, despite increased in modeling precision.

### 3.2. Transducer positioning

The simulation results of the transducer matrix were shown in Tables 2, 3, where the points with the peak AP in the transducer matrix were selected as the center of the transducer. The calculation of the transducer matrix AP at a single frequency with a 256-grid model required approximately 490.5 h. Figure 5A presented the results of the AP comparison between the positioned transducer and the transducer at different angles corresponding to the plane directions where the target region was located. The AP of transducer center on the coronal axis ( XY,YZ=0° ) was closest to the positioned transducer in each model, with ratios at each frequency was 250 kHz: 84.57% ± 9.67%, 500 kHz: 80.80% ± 18.42%, 690 kHz: 94.55% ± 3.94%, 1,100 kHz: 89.24% ± 4.96%. The vector angles in each direction of the sonication path were determined based on the transducer positioning results in each precision model, indicating that the incidence angle of sonication changed while the thickness of the skull layer affected by model precision. The variation in the angle between the positioned sonication path and the coronal and transverse planes was found to be insignificant with low-frequency. Notably, as the frequency increases, the angle variation with different precisions becomes more pronounced. It was found that the angle to the coronal plane obtained at all precision and frequency models did not exceed 10° and 15° to the transverse plane. The stimulus distances between the positioned center and the target region were observed to have a range of 27 to 31 mm. Additionally, our findings, as shown in Table 3, indicated that this distance remained stable and did not significantly vary with increasing model precision.

**Table 2:** Simulation results at different frequencies.

Parameters		Group 1	Group 2	Group 3	Group 4
Frequency (kHz)		250 kHz	500 kHz	690 kHz	1,100 kHz
Angles with transverse plane; sagittal plane; coronal plane		9.88° ± 3.03° 78.44° ± 3.95° 5.64° ± 2.51°	11.46° ± 3.45° 76.42° ± 2.56° 6.67° ± 2.40°	5.01° ± 5.37° 83.44° ± 6.14° 3.75° ± 3.73°	6.19° ± 6.44° 82.93° ± 7.84° 2.84° ± 4.92°
Stimulus distance (mm)		27.25 ± 0.28	28.81 ± 1.78	30.31 ± 0.47	28.38 ± 1.62
Deviation*	Length (mm) Width (mm) Offset (°) AP (%)	23.66 ± 4.70(43.87% ± 11.19%) 4.62 ± 3.04(37.05% ± 19.72%) 3.20 ± 0.93 47.97 ± 23.41	22.60 ± 10.75(52.41% ± 21.91%) 2.49 ± 1.97(28.00% ± 21.95%) 2.28 ± 5.51 48.51 ± 23.16	28.80 ± 2.71(55.76% ± 7.00%) 1.61 ± 0.96(18.65% ± 13.02%) 2.11 ± 6.10 47.52 ± 16.41	21.96 ± 8.59(43.30% ± 10.34%) 1.72 ± 1.59(15.76% ± 13.72%) 1.72 ± 2.97 47.73 ± 7.54
Peak temperature (°C)		39.10 ± 0.35	39.95 ± 1.43	41.44 ± 2.67	38.64 ± 0.11

* The deviation captured the error associated with the FWHM measurements in both the water medium and the sonication that penetrated the skull.

**Table 3:** Simulation results with different precision models.

Parameters		Group 1	Group 2	Group 3
Simulation domain (grid)		128 × 128 × 128	210 × 210 × 210	256 × 256 × 256
Angles with transverse plane; sagittal plane; coronal plane (°)		11.11° ± 2.51° 76.20° ± 2.28° 8.01° ± 0.60°	5.56° ± 6.22° 83.39° ± 6.15° 3.00° ± 2.01°	7.73° ± 4.89° 81.33° ± 6.04° 3.18° ± 4.08°
Stimulus distance (mm)		28.56 ± 1.22	28.84 ± 2.15	28.67 ± 1.65
FWHM at transverse plane	Length (mm) Width (mm) Offset (°)	25.62 ± 1.89 9.41 ± 3.56 12.50 ± 2.72	28.17 ± 6.84 6.79 ± 2.25 −1.55 ± 6.95	22.90 ± 11.2 5.12 ± 1.40 6.56 ± 5.12
Deviation*	Length (mm) Width (mm) Offset (°) AP (%)	29.93 ± 2.33(53.87% ± 2.02%) 4.32 ± 2.64(33.07% ± 24.97%) 4.20 ± 1.04 52.29 ± 13.27	20.64 ± 7.44(41.73% ± 12.30%) 1.21 ± 1.24(13.76% ± 14.89%) −1.22 ± 3.75 31.62 ± 10.46	24.47 ± 6.13(54.54% ± 17.96%) 2.40 ± 1.42(31.14% ± 16.76%) 4.00 ± 3.51 59.89 ± 9.21
Peak temperature (°C)		38.62 ± 0.18	40.83 ± 2.25	38.90 ± 1.54
Matrix points		213	977 (2 points intracranial)	1811 (9 points intracranial)
Distance between matrix and target region (mm)		Average: 29.21; Min: 24.49; Max: 31.56	Average: 27.83; Min: 20.86; Max: 30.82	Average: 28.64; Min: 22.65; Max: 30.90
Computational time (per frequency)		4.8 h	143 h	490.5 h

* The deviation captured the error associated with the FWHM measurements in both the water medium and the sonication that penetrated the skull.

### 3.3. Effects of FWHM

**Figure 6:**
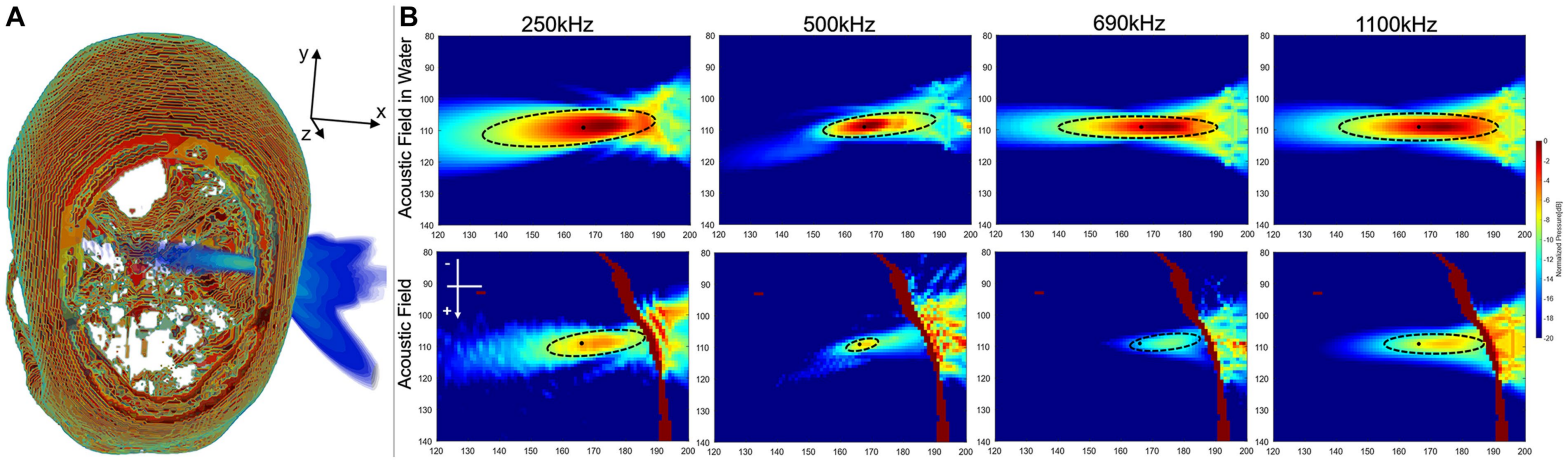
Results of sonication penetrating the skull. **(A)** Spatial field of sonication penetrating the skull. **(B)** The acoustic field in the transverse plane was analyzed both in the water medium surrounding the target region and within the skull medium in the high-precision model of the 256×256×256 domain.

The results in the 256×256×256 domain were 250 kHz: MI 0.19 and spatial-peak temporal-average intensity ( $I_{\mathrm{SPTA}}$ ) 630.24 mW/cm^2^, 500 kHz: MI 0.13 and $I_{\mathrm{SPTA}}$ 487.26 mW/cm^2^, 690 kHz: MI 0.11 and $I_{\mathrm{SPTA}}$ 487.26 mW/cm^2^, 1,100 kHz: MI 0.02 and $I_{\mathrm{SPTA}}$ 175.49 mW/cm^2^. The acoustic field results of sonication penetrating the skull in the 256×256×256 domain were shown in Figure 6. Figure 6A illustrated the spatial acoustic field under sonication at 500 kHz, while Figure 6B provided a comparison of the acoustic field under sonication at each frequency after penetrating through water and experiencing attenuation in the skull. In this study, the range of FWHM contours was analyzed as an indicator of the most effective region of sonication stimulation. Tables 2, 3 presented the length-width measurements of the FWHM at each frequency and precision model, as well as the attenuation observed after penetrating the skull. The results indicated that as the skull layer was refined, the attenuation of the FWHM diminished accordingly. Notably, the 128-grid model in twofold precision yielded highly comparable results to the FWHM deviation observed in the 256-grid model while also improving computational time efficiency. However, no significant changes were observed in FWHM deviation and AP attenuation across different frequencies. The generated FWHM in the transverse plane of the target region under sonication stimulation at each frequency can effectively cover the treatment range for tumors with a diameter approximately within the 30 mm range.

### 3.4. Effects of thermal effect

**Figure 7:**
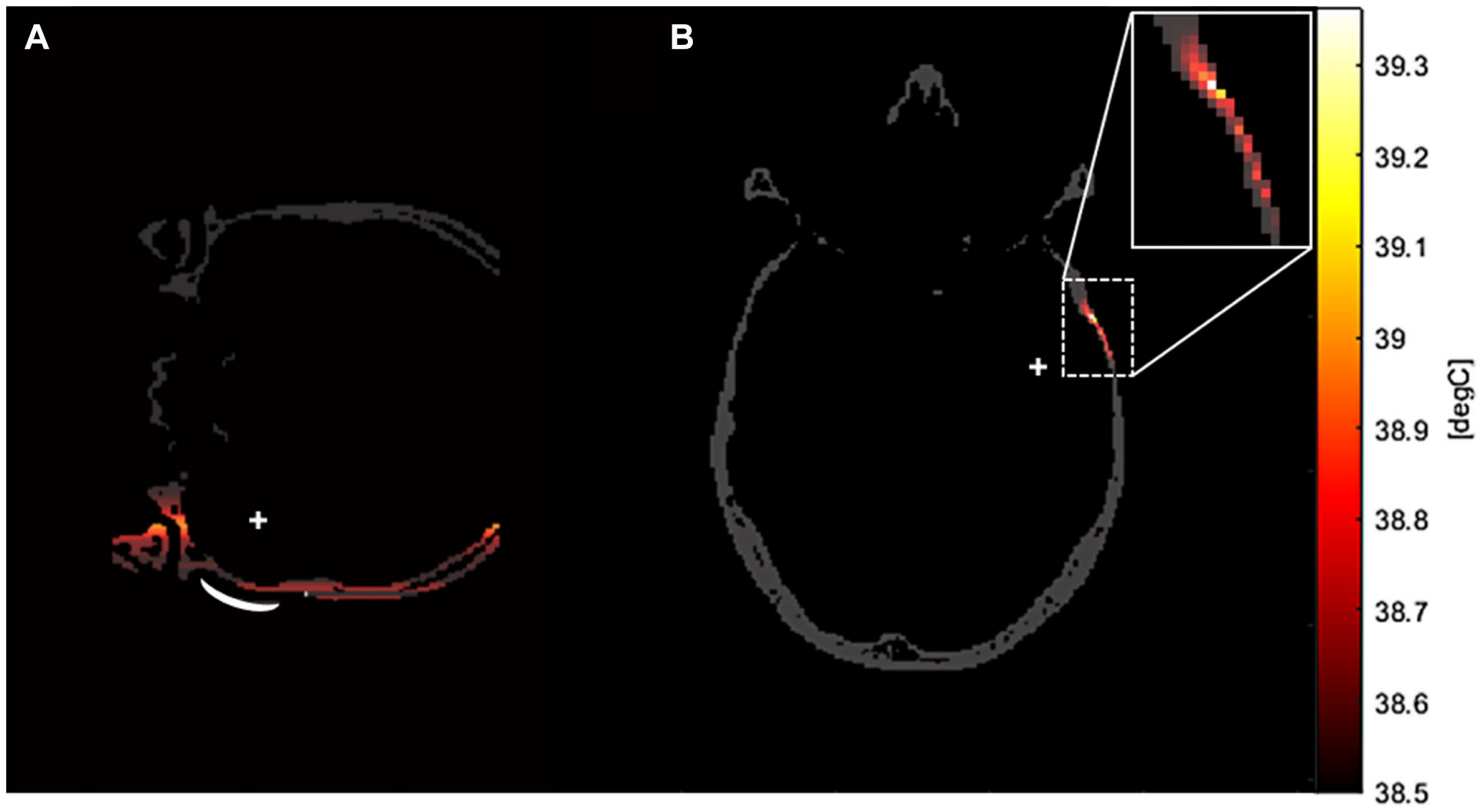
The temperature field of sonication-induced thermal effects in the transverse plane of the target region in the high-precision model. This thermal simulation was conducted in a 256×256×256 domain, with a stimulation frequency of 500 kHz. As depicted in the example graphs, the majority of the heat generation in tFUS occurred in the skull layer across all frequency models. **(A)** Was the coronal plane of the temperature field. **(B)** Was the transverse plane of the temperature field.

The peak temperatures generated through sonication stimulation at various frequencies were depicted in Tables 2, 3, corresponding to distinct precision models. Notably, in the original precision model, the peak temperature produced by sonication stimulation at 690 kHz was measured at 43.73°C. The temperature field of sonication-induced thermal effects at the target region was shown in Figures 7A,B, indicating that most of the heat generated in the skull layer and did not achieve the denaturation value of 47°C that lead to cancellous osteonecrosis. As previously mentioned (Dickson and Calderwood, 1980; Augustin et al., 2012; Darrow, 2019), low-intensity tFUS has been observed to have no detrimental effects on brain tissue and its effects on brain tumors are not attributed to thermal effects.

## 4. Discussion

In this study, we presented a novel framework for tFUS simulation aimed at treating temporal lobe tumors by using the cavitation effect based on the latest advances in neuromodulation research. In this paper, a new RP method was proposed to position the sonication transducer. To our knowledge, we were the first to build a framework for sonication treatment based on numerical simulation of the RP method for transducer positioning to ensure safety under sonication stimulation through the thermal effects and matrix selection of transducer positioning among all known tFUS studies. The results of the sonication-induced temperature field analysis suggested that the influence of tFUS on tumors was independent of thermal effects. Obtaining the optimal range of ultrasonic stimulation pathways based on the selected transducer parameters. Significantly, we incorporate the conventional algorithm in simulating the transducer peak pressure, setting our study apart from earlier studies that rely on arbitrary transducer positioning. The results demonstrated the superior effectiveness of our positioned transducer in tFUS applications.

So many researchers were attracted by tFUS due to its noninvasiveness, high resolution, and ability to focus deeply. However, different stimulation parameters had been used in sonication neuromodulation studies leading to diverse results, where the focus was hard to determine after penetrating the skull. In this study, a model was constructed based on the CT images of the brain after binarization. Next, a simulation framework was built using the k-Wave toolbox for AP simulation based on the actual transducer probe properties. And the accuracy of positioning was verified by the control group simulation of each directional angle. Finally, the acoustic field and temperature fields of sonication-induced thermal effects were performed. The results demonstrated that the positioned sonication transducer provided a safe and effective maximum effect. Furthermore, this framework presented a new approach to positioning error control for practical tFUS neuromodulation, which will advance the application of tFUS in ultrasound neuromodulation. It has been suggested that when time is limited for precise positioning, using a twofold precision model to calculate the optimal stimulus path can also yield positive results for stimulation.

In porous media, the propagation of ultrasonic waves becomes more intricate due to the non-uniformity inherent in the medium. Consequently, strictly relying on linear assumptions for simulation may not be entirely valid. To address this concern, we constructed a grid domain to discretize the inhomogeneous skull, aiming to achieve the most accurate simulation of the complex acoustic field possible. To ensure precision, we calculated the porosity by utilizing CT image data, enabling us to describe the speed of sound and attenuation within the medium. While indirect assumptions were made regarding pore structure, pore spacing, and pore shape, our approach aimed to approximate these characteristics to the best of our ability. In an attempt to conduct more precise calculations, we tried to perform a higher resolution model. However, due to a substantial increase in computational time from 4.8 h to 490.5 h when transitioning from the twofold precision model to the original precision model, this computational cost dictated the upper limit of resolution within the scope of this study.

In similar tFUS studies, Park et al. compared duty cycle and sonication duration by used a single exploration of ultrasound parameters, focusing on assessing the feasibility of ultrasound framework (Park et al., 2022). Furthermore, we focused on numerical simulation results of the effect of sonication beam parameters on the intracranial target region. Due to the time-consuming nature of the transducer positioning accurately (taking more than 490.5 h for high-precision models), most studies (Beisteiner et al., 2019; Grudzenski et al., 2022; Koh et al., 2022) explored the effect of tFUS by arbitrary choosing a region as the transducer center, which may result in the peak pressure region of stimulation being off-target. The arbitrary transducer positions also explored the attenuation of ultrasound with a high degree of contingency for non-uniform skull layer. The results (Figure 4) illustrated that the contingency for the simulation results in skull not only pertained to the position of the transducer but also to the chosen target region for different precision models.

Previous studies by Nelson and Nunneley (1998) and Park et al. (2022) had investigated the safety of sonication stimulation based on thermal variation, using duration of stimulation versus depth. In this study, we took a different approach by performing real-time detection of the intracranial temperature of each precision model, using assigned thermal diffusion coefficients (i.e., specific heat capacity and thermal conductivity) to the cerebrospinal fluid and skull layer, indicated that most of the heat generated within short-term sonication stimulation was absorbed by the skull layer. This was in line with the study by Augustin et al. (2012) and Dickson and Calderwood (1980), where the peak temperature generated by sonication did not exceed the threshold for damage to the skull layer, further supporting the overall safety of tFUS.

One limitation of our study was the time-consuming nature of the computation, which restricted the number of frequency controls we could perform. The transducer positioning procedure at a frequency of 1 mm/grid precision model took an average of 490.5 h accelerated by NVIDIA GeForce RTX 2070 GPU. Additionally, ethical considerations hindered the application of tFUS in clinical, making it impossible for us to prove its effectiveness in a practical setting. Nonetheless, we took into account all evaluable results as much as possible under the simulated conditions at all current hotspot sets of frequencies in our study.

Based on our findings, the ability to modulate the directional angle of the sonication stimulus within ±15° in the coronal plane resulted in an average computation time of 24.62% when employing the high-precision model, which required 120.76 h of computation. Additionally, compromising the model precision led to a calculation time of 63.9% of the original duration, allowing completion of the calculations in 184 min using a model with reduced granularity. These results demonstrate the potential for approximating the clinical environment and pave the way for future investigations into the feasibility of utilizing lower granularity models for calculations.

In the future, this research will be further enriched with the following methods. Firstly, this study will be further enriched by collecting additional data to validate the potential effects of inter-individual differences on transducer positioning. Secondly, the method will be used in animal models (i.e., rats, rabbits) for simulation to demonstrate the feasibility of this ultrasound framework, with the expectation of achieving positive results for any size of skull. Successively, the parameters in this study will be pinned down for application to animal experiments through actual sonication output. Finally, based on the combination of existing research (Kim et al., 2018; Mohammadjavadi et al., 2019; Bobola et al., 2020; Wang et al., 2021), including error analysis of measurement results under actual sonication stimulation and simulation results. The method will be evolved into a practical tFUS framework system, ultimately serving clinicians.

## 5. Conclusion

We have proposed a framework for predicting tFUS treatment using the k-Wave toolbox, which determines the optimal treatment position through a specialized transducer positioning method (RP method) and explores the distribution of the sonication beam at different frequencies using brain CT images. Notably, we have demonstrated the feasibility of this tFUS framework by predicting the acoustic temperature field of sonication-induced thermal effects. The angle of sonication stimulation path in both the coronal and transverse planes plays a critical role in determining the appropriate program for transducer positioning during the preliminary stages. Successively, we verify the effectiveness of the transducer positioning by the results of the control group at each directional angle. This framework proposed offers an accurate and effective method for numerical prediction of brain tumors treatment using sonication. In the future, as we continue to refine our method, this framework may eventually play a pivotal role in clinical settings for precise treatment of brain tumors.

## Data availability statement

The raw data supporting the conclusions of this article will be made available by the authors, without undue reservation.

## Ethics statement

The study involving human participants was approved by the Ethics Committee of Shandong First Medical University Affiliated Cancer Hospital (Approval ID: SDTHEC2023002013) and the written informed consent was waived in accordance with the national legislation and the institutional requirements.

## Author contributions

PG: Conceptualization, Investigation, Methodology, Software, Validation, Visualization, Writing – original draft. YS: Investigation, Writing – review & editing. GZ: Formal analysis, Writing – review & editing. CL: Conceptualization, Methodology, Writing – review & editing. LW: Data curation, Funding acquisition, Writing – review & editing.

## References

[ref1] Armstrong S. A. Jafary R. Forsythe J. S. Gregory S. D. (2023). Tissue-mimicking materials for ultrasound-guided needle intervention phantoms: a comprehensive review. Ultrasound Med. Biol. 49, 18–30. doi: 10.1016/j.ultrasmedbio.2022.07.016 36210247

[ref2] Augustin G. Zigman T. Davila S. Udilljak T. Staroveski T. Brezak D.. (2012). Cortical bone drilling and thermal osteonecrosis. Clin. Biomech. 27, 313–325. doi: 10.1016/j.clinbiomech.2011.10.010, PMID: 22071428

[ref3] Beisteiner R. Matt E. Fan C. Baldysiak H. Schönfeld M. Philippi Novak T.. (2019). Transcranial pulse stimulation with ultrasound in Alzheimer's disease-a new navigated focal brain therapy. Adv. Sci. (Weinh). 7:1902583. 32042569 10.1002/advs.201902583 PMC7001626

[ref4] Bjørnø L. (2010). Introduction to nonlinear acoustics. Phys. Procedia 3, 5–16. doi: 10.1016/j.phpro.2010.01.003 PMC3070311 21472037

[ref5] Bobola M. S. Chen L. Ezeokeke C. K. Olmstead T. A. Nguyen C. Sahota A.. (2020). Transcranial focused ultrasound, pulsed at 40 Hz, activates microglia acutely and reduces Aβ load chronically, as demonstrated *in vivo*. Brain Stimul. 13, 1014–1023. doi: 10.1016/j.brs.2020.03.016, PMID: 32388044 PMC7308193

[ref6] Bystritsky A. Korb A. S. Douglas P. K. Cohen M. S. Melega W. P. Mulgaonkar A. P.. (2011). A review of low-intensity focused ultrasound pulsation. Brain Stimul. 4, 125–136. doi: 10.1016/j.brs.2011.03.007, PMID: 21777872

[ref7] Cain J. A. Spivak N. M. Coetzee J. P. Crone J. S. Johnson M. A. Lutkenhoff E. S.. (2022). Ultrasonic deep brain neuromodulation in acute disorders of consciousness: a proof-of-concept. Brain Sci. 12:428. doi: 10.3390/brainsci12040428, PMID: 35447960 PMC9032970

[ref8] Cappon D. den Boer T. Jordan C. Yu W. Metzger E. Pascual-Leone A. (2022). Transcranial magnetic stimulation (TMS) for geriatric depression. Ageing Res. Rev. 74:101531. doi: 10.1016/j.arr.2021.101531, PMID: 34839043 PMC8996329

[ref9] Cho H. Lee H.-Y. Han M. Choi J.-R. Ahn S. Lee T.. (2016). Localized Down-regulation of P-glycoprotein by focused ultrasound and microbubbles induced blood-brain barrier disruption in rat brain. Sci. Rep. 6:31201. doi: 10.1038/srep31201, PMID: 27510760 PMC4980618

[ref10] Connor C.W. Simulation methods and tissue property models for non-invasive transcranial focused ultrasound surgery. Doctor’s Thesis, Massachusetts Institute of Technology, Massachusetts (2005).

[ref11] Darrow D. P. (2019). Focused ultrasound for neuromodulation. Neurotherapeutics 16, 88–99. doi: 10.1007/s13311-018-00691-3, PMID: 30488340 PMC6361056

[ref12] Dickson J. A. Calderwood S. K. (1980). Temperature range and selective sensitivity of tumors to hyperthermia: a critical review. Ann. N. Y. Acad. Sci. 335, 180–205. doi: 10.1111/j.1749-6632.1980.tb50749.x, PMID: 6931518

[ref13] Duck F.A. Physical properties of tissue. A comprehensive reference book, London, England: Academic Press (1990).

[ref14] Grisey A. Heidmann M. Letort V. Lafitte P. Yon S. (2016). Influence of skin and subcutaneous tissue on high-intensity focused ultrasound beam: experimental quantification and numerical modeling. Ultrasound Med. Biol. 42, 2457–2465. doi: 10.1016/j.ultrasmedbio.2016.06.013, PMID: 27471120

[ref15] Grudzenski S. Heger S. de Jonge A. Schipp J. Dumont E. Larrat B.. (2022). Simulation, implementation and measurement of defined sound fields for blood-brain barrier opening in rats. Ultrasound Med. Biol. 48, 422–436. doi: 10.1016/j.ultrasmedbio.2021.10.003, PMID: 34863589

[ref16] Huh H. Park T. Y. Seo H. Han M. Jung B. Choi H. J.. (2020). A local difference in blood–brain barrier permeability in the caudate putamen and thalamus of a rat brain induced by focused ultrasound. Sci. Rep. 10:19286. doi: 10.1038/s41598-020-76259-z, PMID: 33159137 PMC7648079

[ref17] Iglesias A. H. (2020). Transcranial magnetic stimulation as treatment in multiple neurologic conditions. Curr. Neurol. Neurosci. Rep. 20:1. doi: 10.1007/s11910-020-1021-0, PMID: 32020300

[ref18] Ilovitsh T. Ilovitsh A. Foiret J. Caskey C. F. Kusunose J. Fite B. Z.. (2018). Enhanced microbubble contrast agent oscillation following 250 kHz insonation. Sci. Rep. 8:16347. doi: 10.1038/s41598-018-34494-5, PMID: 30397280 PMC6218550

[ref19] Jing B. Lindsey B. D. (2021). Effect of skull porous trabecular structure on transcranial ultrasound imaging in the presence of elastic wave mode conversion at varying incidence angle. Ultrasound Med. Biol. 47, 2734–2748. doi: 10.1016/j.ultrasmedbio.2021.05.010, PMID: 34140169

[ref20] Kim S. Kim H. Shim C. Lee H. J. (2018). Improved target specificity of transcranial focused ultrasound stimulation (TFUS) using double-crossed ultrasound transducers. Annu. Int. Conf. IEEE Eng. Med. Biol. Soc. 2679–2682. doi: 10.1109/EMBC.2018.8512812 30440958

[ref21] Kinoshita M. McDannold N. Jolesz F. A. Hynynen K. (2006). Noninvasive localized delivery of Herceptin to the mouse brain by MRI-guided focused ultrasound-induced blood–brain barrier disruption. Proc. Natl. Acad. Sci. U. S. A. 103, 11719–11723. doi: 10.1073/pnas.0604318103, PMID: 16868082 PMC1544236

[ref22] Klomjai W. Katz R. Lackmy-Vallée A. (2015). Basic principles of transcranial magnetic stimulation (TMS) and repetitive TMS (rTMS). Ann. Phys. Rehabil. Med. 58, 208–213. doi: 10.1016/j.rehab.2015.05.005 26319963

[ref23] Koh H. Park T. Y. Chung Y. A. Lee J. H. Kim H. (2022). Acoustic simulation for transcranial focused ultrasound using GAN-based synthetic CT. IEEE J. Biomed. Health Inform. 26, 161–171. doi: 10.1109/JBHI.2021.3103387, PMID: 34388098

[ref24] Konofagou E. E. Tung Y. S. Choi J. Deffieux T. Baseri B. Vlachos F. (2012). Ultrasound-induced blood-brain barrier opening. Curr. Pharm. Biotechnol. 13, 1332–1345. doi: 10.2174/138920112800624364, PMID: 22201586 PMC4038976

[ref25] Krishna V. Sammartino F. Rezai A. (2018). A review of the current therapies, challenges, and future directions of transcranial focused ultrasound technology: advances in diagnosis and treatment. JAMA Neurol. 75, 246–254. doi: 10.1001/jamaneurol.2017.3129 29228074

[ref26] Kubanek J. (2018). Neuromodulation with transcranial focused ultrasound. Neurosurg. Focus. 44:E14. doi: 10.3171/2017.11.FOCUS17621, PMID: 29385924 PMC5927579

[ref27] Lee W. Chung Y. A. Jung Y. Song I. U. Yoo S. S. (2016). Simultaneous acoustic stimulation of human primary and secondary somatosensory cortices using transcranial focused ultrasound. BMC Neurosci. 17:68. doi: 10.1186/s12868-016-0303-6, PMID: 27784293 PMC5081675

[ref28] Lee W. Kim H. Jung Y. Song I. U. Chung Y. A. Yoo S. S. (2015). Image-guided transcranial focused ultrasound stimulates human primary somatosensory cortex. Sci. Rep. 5:8743. doi: 10.1038/srep08743, PMID: 25735418 PMC4348665

[ref29] Legon W. Ai L. Bansal P. Mueller J. K. (2018). Neuromodulation with single-element transcranial focused ultrasound in human thalamus. Hum. Brain Mapp. 39, 1995–2006. doi: 10.1002/hbm.23981, PMID: 29380485 PMC6866487

[ref30] Lescrauwaet E. Vonck K. Sprengers M. Raedt R. Klooster D. Carrette E.. (2022). Recent advances in the use of focused ultrasound as a treatment for epilepsy. Front. Neurosci. 16:886584. doi: 10.3389/fnins.2022.886584, PMID: 35794951 PMC9251412

[ref31] Marder K. G. Barbour T. Ferber S. Idowu O. Itzkoff A. (2022). Psychiatric applications of repetitive transcranial magnetic stimulation. Focus (Am. Psychiatr. Publ). 20, 8–18. doi: 10.1176/appi.focus.20210021, PMID: 35746935 PMC9063593

[ref32] Maresca D. Lakshmanan A. Abedi M. Bar-Zion A. Farhadi A. Lu G. J.. (2018). Biomolecular ultrasound and Sonogenetics. Annu. Rev. Chem. Biomol. Eng. 9, 229–252. doi: 10.1146/annurev-chembioeng-060817-084034, PMID: 29579400 PMC6086606

[ref33] Min B. K. Bystritsky A. Jung K. I. Fischer K. Zhang Y. Maeng L. S.. (2011). Focused ultrasound-mediated suppression of chemically-induced acute epileptic EEG activity. BMC Neurosci. 12:23. doi: 10.1186/1471-2202-12-23, PMID: 21375781 PMC3061951

[ref34] Mohammadjavadi M. Ye P. P. Xia A. Brown J. Popelka G. Pauly K. B. (2019). Elimination of peripheral auditory pathway activation does not affect motor responses from ultrasound neuromodulation. Brain Stimul. 12, 901–910. doi: 10.1016/j.brs.2019.03.005, PMID: 30880027 PMC6592746

[ref35] Mueller J. K. Ai L. Bansal P. Legon W. (2017). Numerical evaluation of the skull for human neuromodulation with transcranial focused ultrasound. J. Neural Eng. 14:066012. doi: 10.1088/1741-2552/aa843e 28777075

[ref36] Nelson D. A. Nunneley S. A. (1998). Brain temperature and limits on transcranial cooling in humans: quantitative modeling results. Eur. J. Appl. Physiol. Occup. Physiol. 78, 353–359. doi: 10.1007/s004210050431, PMID: 9754976

[ref37] Park T. Y. Jeong J. H. Chung Y. A. Yeo S. H. Kim H. (2022). Application of subject-specific helmets for the study of human visuomotor behavior using transcranial focused ultrasound: a pilot study. Comput. Methods Prog. Biomed. 226:107127. doi: 10.1016/j.cmpb.2022.107127, PMID: 36126434

[ref38] Park C. Y. Seo H. Lee E.-H. Han M. Choi H. Park K.-S.. (2021). Verification of blood-brain barrier disruption based on the clinical validation platform using a rat model with human skull. Brain Sci. 11:1429. doi: 10.3390/brainsci11111429, PMID: 34827428 PMC8615862

[ref39] Rzechorzek N. M. Thrippleton M. J. Chappell F. M. Mair G. Ercole A. Cabeleira M.. (2022). A daily temperature rhythm in the human brain predicts survival after brain injury. Brain 145, 2031–2048. doi: 10.1093/brain/awab466 35691613 PMC9336587

[ref40] Schwenke M. Georgii J. Preusser T. (2017). Fast numerical simulation of focused ultrasound treatments during respiratory motion with discontinuous motion boundaries. IEEE Trans. Biomed. Eng. 64, 1455–1468. doi: 10.1109/TBME.2016.2619741, PMID: 28541191

[ref41] Servick K. (2020). Hope grows for targeting the brain with ultrasound. Science 368, 1408–1409. doi: 10.1126/science.368.6498.1408, PMID: 32586996

[ref42] Shen Z. Y. Shen E. Diao X. H. Bai W. K. Zeng M. X. Luan Y. Y.. (2014). Inhibitory effects of subcutaneous tumors in nude mice mediated by low-frequency ultrasound and microbubbles. Oncol. Lett. 7, 1385–1390. doi: 10.3892/ol.2014.1934, PMID: 24765142 PMC3997662

[ref43] Slegers R. J. Blumcke I. (2020). Low-grade developmental and epilepsy associated brain tumors: a critical update 2020. Acta Neuropathol. Commun. 8:27. doi: 10.1186/s40478-020-00904-x, PMID: 32151273 PMC7063704

[ref44] Slezak C. Flatscher J. Slezak P. (2022). A comparative feasibility study for transcranial extracorporeal shock wave therapy. Biomedicine 10:1457. doi: 10.3390/biomedicines10061457, PMID: 35740477 PMC9219950

[ref45] Svider P. F. Blasco M. A. Raza S. N. Shkoukani M. Sukari A. Yoo G. H.. (2017). Head and neck Cancer. Otolaryngol. Head Neck Surg. 156, 10–13. doi: 10.1177/0194599816674672, PMID: 28045631

[ref46] Treeby B. E. Cox B. T. (2010). K-wave: MATLAB toolbox for the simulation and re-construction of photoacoustic wave fields. J. Biomed. Opt. 15:021314. doi: 10.1117/1.3360308, PMID: 20459236

[ref47] Vargo M. (2011). Brain tumor rehabilitation. Am. J. Phys. Med. Rehabil. 90, 50–62. 10.1097/PHM.0b013e31820be31f 21765264

[ref48] Venkatesh H. S. Johung T. B. Caretti V. Noll A. Tang Y. Nagaraja S.. (2015). Neuronal activity promotes glioma growth through Neuroligin-3 secretion. Cells 161, 803–816. doi: 10.1016/j.cell.2015.04.012, PMID: 25913192 PMC4447122

[ref49] Venkatesh H. Monje M. (2017). Neuronal activity in ontogeny and oncology. Trends Cancer. 3, 89–112. doi: 10.1016/j.trecan.2016.12.008, PMID: 28718448 PMC5518622

[ref50] Wang J. Li G. Deng L. Mamtilahun M. Jiang L. Qiu W.. (2021). Transcranial focused ultrasound stimulation improves neurorehabilitation after middle cerebral artery occlusion in mice. Aging Dis. 12, 50–60. doi: 10.14336/AD.2020.0623, PMID: 33532127 PMC7801287

[ref51] Xu L. Lee W. Rotenberg A. Böhlke M. Yoon K. Yoo S. S. (2020). Localized disruption of blood albumin–phenytoin binding using transcranial focused ultrasound. Ultrasound Med. Biol. 46, 1986–1997. doi: 10.1016/j.ultrasmedbio.2020.04.011, PMID: 32402673

[ref52] Yan Y. Chen Y. Liu Z. Cai F. Niu W. Song L.. (2021). Brain delivery of curcumin through low-intensity ultrasound-induced blood-brain barrier opening via lipid-PLGA Nanobubbles. Int. J. Nanomedicine 16, 7433–7447. doi: 10.2147/IJN.S327737, PMID: 34764649 PMC8575349

[ref53] Yoon K. Lee W. Croce P. Cammalleri A. Yoo S. S. (2018). Multi-resolution simulation of focused ultrasound propagation through ovine skull from a single-element transducer. Phys. Med. Biol. 63:105001. doi: 10.1088/1361-6560/aabe37, PMID: 29658494 PMC5990022

[ref54] Zou J.J. Therapeutic effect of low-intensity focused ultrasound on non-human primate epilepsy model. Master's Thesis, Southern Medical University, Guangzhou (2020).

